# Identification of long noncoding RNAs for the detection of early stage lung squamous cell carcinoma by microarray analysis

**DOI:** 10.18632/oncotarget.14522

**Published:** 2017-01-05

**Authors:** Zule Cheng, Yanan Bai, Ping Wang, Zhenhua Wu, Lin Zhou, Ming Zhong, Qinghui Jin, Jianlong Zhao, Hailei Mao, Hongju Mao

**Affiliations:** ^1^ State Key Laboratory of Transducer Technology, Shanghai Institute of Microsystem and Information Technology, Chinese Academy of Science, Shanghai 200050, China; ^2^ University of Chinese Academy of Sciences, Beijing 100039, China; ^3^ Departments of Anesthesiology and Critical Care Medicine, Zhongshan Hospital, Fudan University, Shanghai 200032, China

**Keywords:** long noncoding RNA, biomarker, diagnosis, early stage lung squamous cell carcinoma, microarray

## Abstract

The aberrant expressions of long noncoding RNAs have been reported in numerous cancers, which have facilitated the cancer diagnosis. However, the expression profile of lncRNAs in early stage lung squamous cell carcinoma has not been well discussed. The present study aimed to examine the expression profile of lncRNAs in early stage lung squamous cell carcinoma and identify lncRNA biomarkers for diagnosis. Through high-throughput lncRNA microarray, we screened thousands of aberrantly expressed lncRNAs and mRNAs in early stage lung squamous cell carcinoma tissues compared to their corresponding adjacent nontumorous tissues. Bioinformatics analyses were used to investigate the functions of aberrantly expressed mRNAs and their associated lncRNAs. After that, in order to identify lncRNA biomarkers for early detection, candidate lncRNA biomarkers were selected based on our established filtering pipeline and validated by real-time quantitative polymerase chain reaction on a total of 63 pairs of tumor samples. Five lncRNAs were finally identified which were able to distinguish early stage tumor and normal samples with high sensitivity (92%) and specificity (83%). These results imply that lncRNAs may be powerful biomarker for early diagnosis.

## INTRODUCTION

Non-small cell lung cancer (NSCLC) is a heterogeneous group of tumors with three subtypes: lung adenocarcinoma (ADC), lung squamous cell carcinoma (LSCC), and large-cell carcinoma. The majority of NSCLC patients is at an advanced disease stage or present with distant metastasis at the time of diagnosis, leaving them with limited treatment options. This delay in diagnosis results in an overall 5-year survival rate of less than 16% [1]. Diagnosing NSCLC earlier would likely reduce overall mortality and increase the rate of remission. As one of the NSCLC subtypes, LSCC accounts for nearly 30% of NSCLC worldwide [2]. Thus, there is considerable interest in developing early detection for LSCC. Biomarkers such as squamous cell carcinoma antigen (SCC-Ag) and CYFRA 21-1 are classic biomarkers commonly used in the diagnoses of LSCCs [3]. However, the current lacking of sufficient diagnostic sensitivity and specificity have limited their usefulness in the early detection of LSCC.

Long noncoding RNAs (lncRNAs) are transcripts longer than 200 nucleotides with little or no protein-coding potential [4]. Dysregulation of lncRNAs is associated with a number of cancer-related processes [5], including epigenetic regulation [6], microRNA silencing [7], DNA damage and cell cycle control [8, 9]. Therefore, lncRNAs have the potential as powerful biomarkers for detecting carcinomas. Some well-studied lncRNAs have been detected in NSCLCs, such as CARLo-5 [10], CCAT2 [11], SOX2OT [12], MEG3 [13] and TUG1 [14]. CARLo-5, CCAT2 and SOX2OT are functioned as onco-lncRNAs and overexpressed in NSCLCs. SOX2OT is a negative prognostic indicator for NSCLC, and the expression level of SOX2OT was found higher in LSCC than ADC [12]. Meanwhile, MEG3 and TUG1 are tumor–suppressor lncRNAs that are downregulated in NSCLCs. With the development of high-throughput technology, increasing number of novel diagnostic and prognostic lncRNA expression signatures are identified, particularly in lung ADC [15, 16]. However, little is known about the lncRNA expression profile during early stage LSCC.

In this work, we used microarray analysis to examine the lncRNA expression profile in early stage LSCC samples against corresponding nontumorous tissue (NT) samples. The signatures of differentially expressed lncRNAs and mRNAs were analyzed. Especially, a novel panel of five lncRNAs was identified which could distinguish early stage LSCC sample from NT samples.

## RESULTS

### Landscape of expressed lncRNAs and mRNAs in LSCC

**Figure 1:**
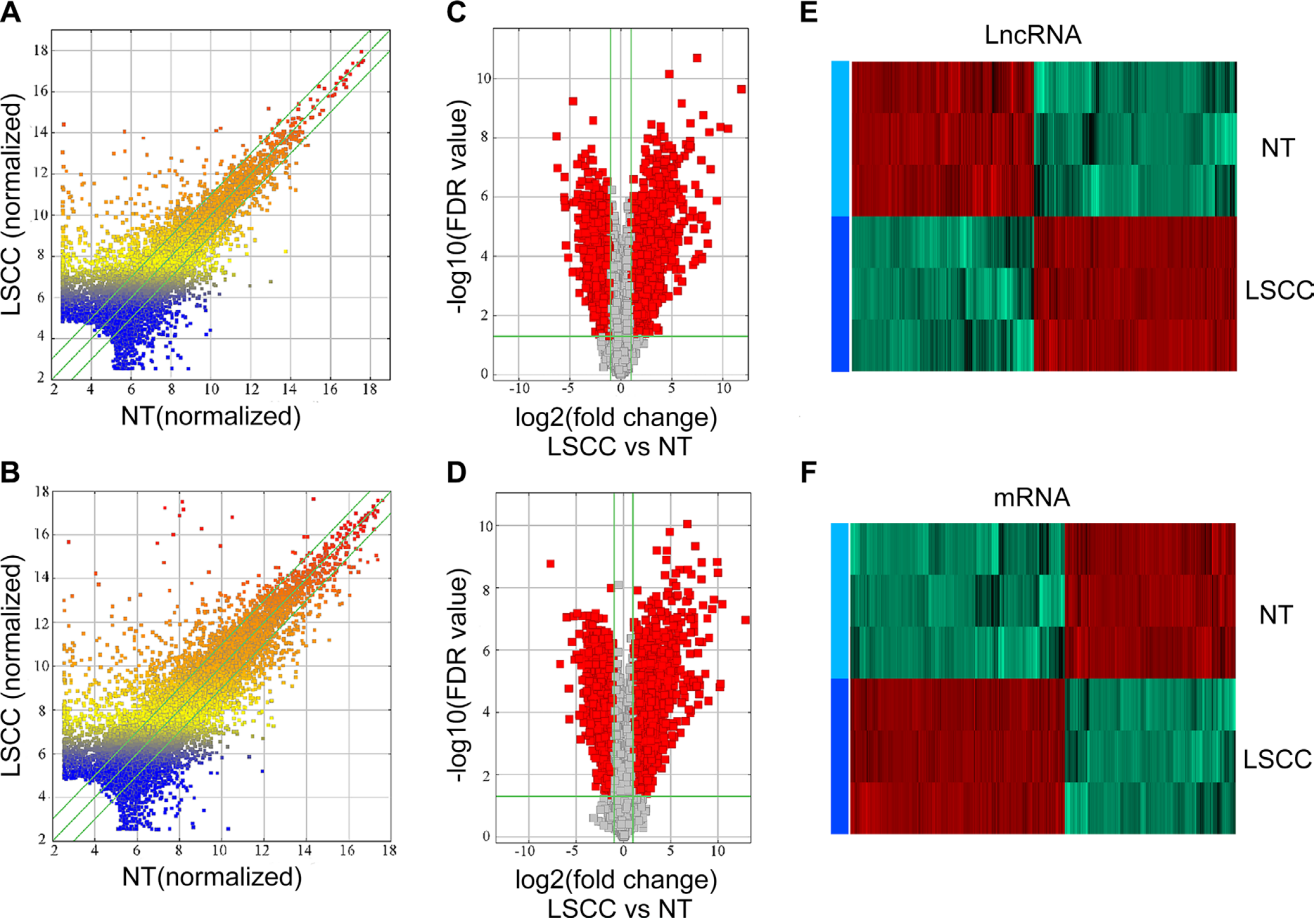
Expression profiles of lncRNAs and mRNAs in LSCCs compared with NT samples (**A** and **B**) Scatter-plot of variation in expression of lncRNAs and mRNAs between early stage LSCC tissues and NT samples. X and Y axes are the mean normalized signal values (log2 scaled). The green lines are fold change lines (the default fold change value given is 2.0). (**C** and **D**) The Volcano plot of differently expressed lncRNAs and mRNAs in LSCC relative to NT samples. The vertical green lines represent 2.0-fold changes up and down and the horizontal green line represents a FDR-value of 0.05. The red points in the plot represent the differentially expressed lncRNAs with statistical significance, X axes are the Fold change values (log2 scaled), Y axes are the FDR-values (log2 scaled). (**E**) and (**F**) Hierarchical clustering analysis of lncRNAs and mRNAs.

In total, 30,586 lncRNAs and 26,109 mRNAs were detected in three pairs of early stage LSCC samples by microarray analysis. Differences of lncRNA and mRNA expression in LSCC samples relative to NT controls are shown in Figure 1A and 1B. A total of 2,668 lncRNAs and 3,728 mRNAs had significantly aberrant expression in all three LSCC samples, as evaluated by volcano plot filtering (Figure 1C and 1D). Of the 2,668 aberrantly expressed lncRNAs in the LSCC samples, 1,407 were upregulated and 1,261 were downregulated. Among the 3,728 differentially expressed mRNAs (DE-mRNA), 2,074 were upregulated and 1,654 were downregulated. Hierarchical clustering analysis (Figure 1E and 1F) resulted in distinct expression signatures of both lncRNAs and mRNAs in the LSCC and NT samples.

### Co-expression analysis of DE-lncRNAs and associated coding genes in LSCC

**Figure 2:**
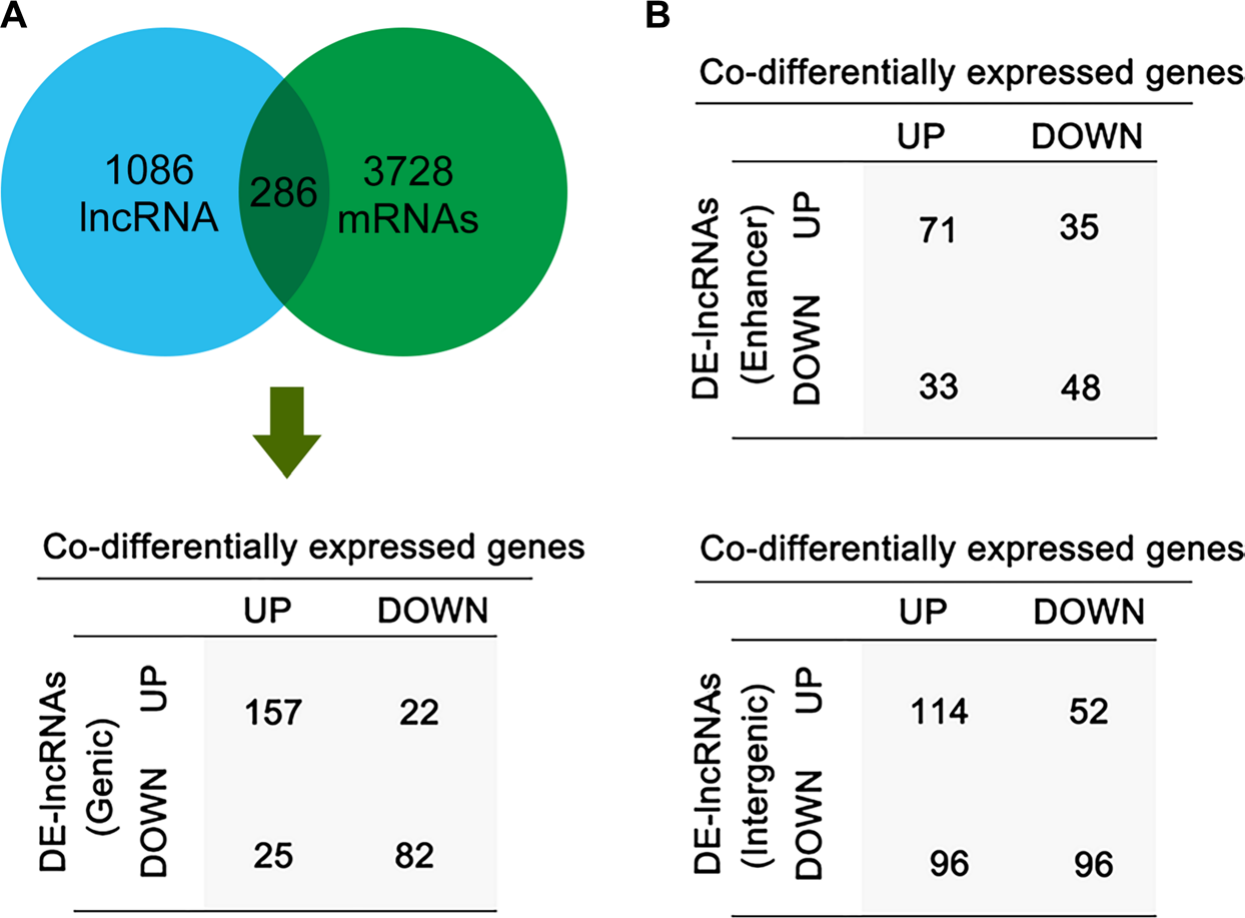
The expression relationship of DE-lncRNAs and their associated coding gene in LSCC (**A**) The analysis of genic lncRNAs and their associated coding genes. The table listed the expression correlation of genic lncRNAs and their associated coding gene. (**B**) The expression correlation of enhance-like lncRNAs (left) and intergenic lncRNA (right) with their nearby coding genes.

Several studies have reported that some lncRNAs can activate or inhibit their associated coding genes by transcriptional interference or chromatin modification [17] [18]. Of the 2,668 DE-lncRNAs, 1,086 genic DE-lncRNAs were localized with their protein coding genes and 286 of these lncRNAs’ associated coding genes were on the list of DE-mRNAs (Figure 2A). Among these 286 coding genes, 83% expressed in the same direction with their corresponding associated lncRNAs, while the rest read in the opposite direction. Furthermore, from the microarray data, we identified 113 enhancer-like lncRNAs and 171 intergenic lncRNAs that were co-aberrantly expressed with their nearby target genes (Figure 2B). We found that some of these lncRNAs have multiple associated target genes. This feature may relate to their locations on the chromosomes.

### Gene ontology and pathway analysis

**Figure 3:**
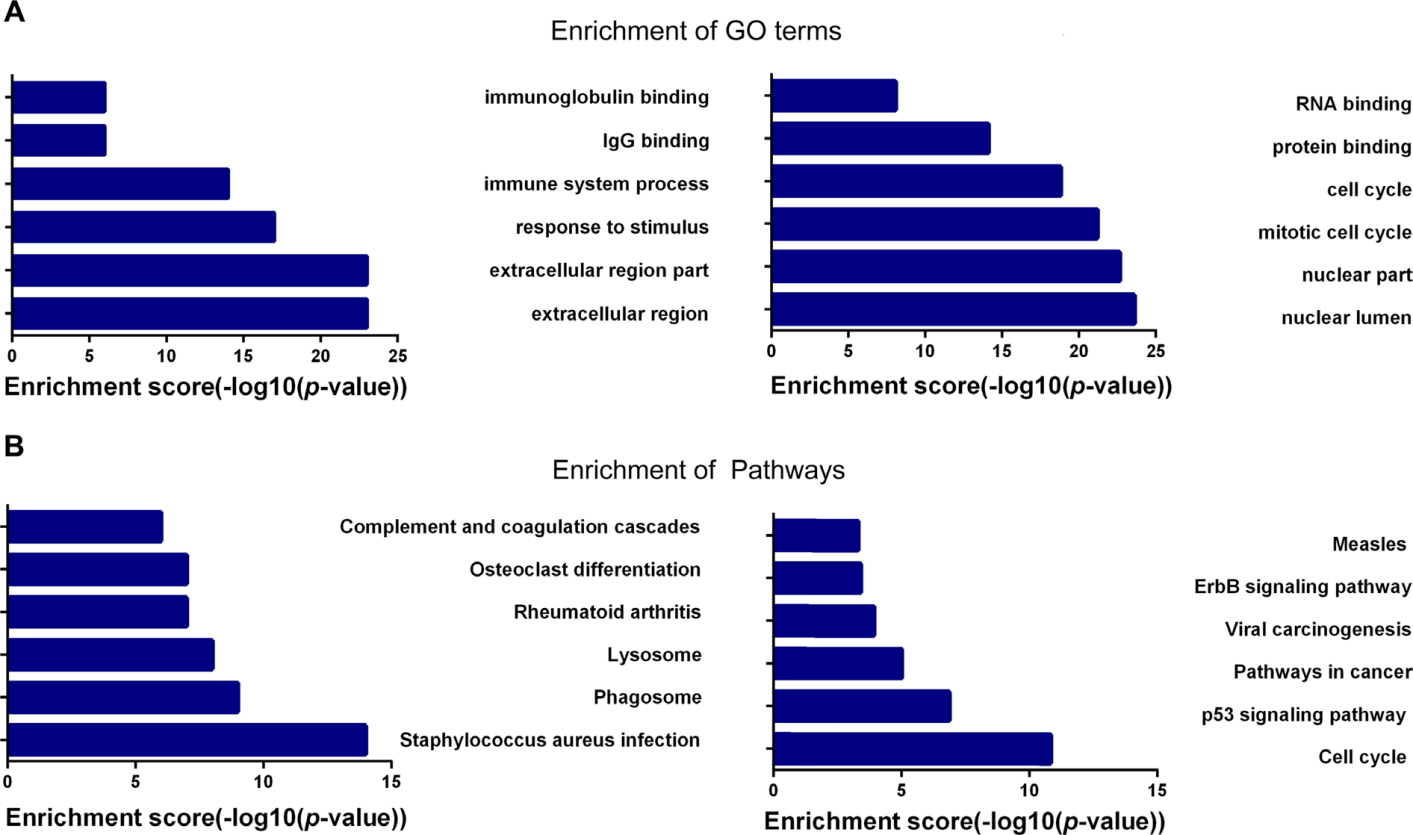
Enrichment analysis of pathways and GO terms for differentially expressed mRNAs The top 6 gene ontology analysis (**A**) and top 6 pathways (**B**) that exhibited significant differences between LSCC and NT samples are listed (left and right panel show the coding genes of downregulated and upregulated, respectively).

To systematically assign putative functions to protein coding genes, we performed GO and KEGG pathway analyses. GO analysis revealed that the upregulated genes were involved in GO terms like nuclear lumen, mitotic cell cycle processes, and protein binding (Figure 3A, right). The downregulated genes were involved in responses to stimuli, extracellular regions, and IgG binding (Figure 3A, left). KEGG pathway analysis was performed by mapping 3,728 DE-mRNAs to KEGG pathways. It was found that upregulated DE-mRNAs were fell disproportionately into several cancer-related pathways (Figure 3B, right). The top three enriched pathways were involved in the cell cycle, p53 signaling and pathways in cancer. ErbB and Wnt signaling pathways also appeared in the top twenty enriched pathways. All these pathways described are closely associated with NSCLCs [19, 20]. By contrast, downregulated mRNAs were disproportionately fell into pathways like *Staphylococcus aureus* infection, phagosomes, and lysosomes, which may be responsible for various infection (Figure 3B, left). Next, we performed the GO and Pathway analysis to the group of DE-lncRNA associated DE-mRNAs which was generated from former analysis. We found these DE-mRNAs were enriched in pathways like Cell cycle, Transcriptional misregulation in cancer and Choline metabolism in cancer (Supplementary Tables 1 and 2). We speculated that some DE-lncRNAs might be either directly or indirectly involved in these pathways.

### Evaluate the diagnostic potential of lncRNAs in early stage LSCC

**Figure 4:**
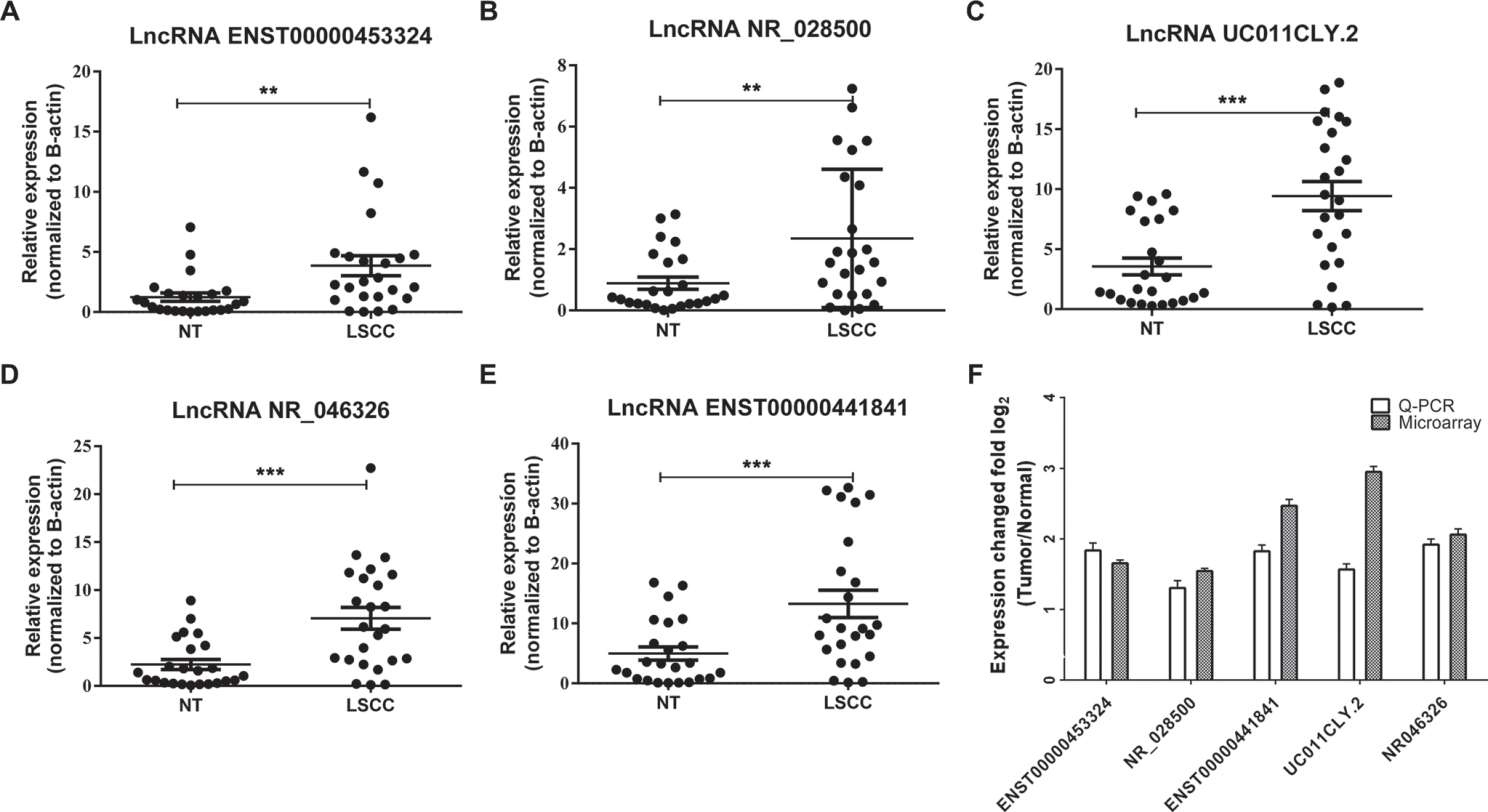
RT-qPCR validation reveals significant lncRNA expression differences in LSCC samples compare to the adjacent normal samples (**A–E**) The expression level of five selected lncRNAs in 24 LSCC samples against corresponding adjacent NT samples. All the differences of five lncRNAs between LSCC samples and NT samples were significant. (**F**) Comparison of the expression change fold of five candidate lncRNAs in three paired patients’ microarray results and 24 paired patients’ qPCR results. The heights of the columns in the chart represent the mean expression value of log2 fold changes (tumor/normal) for each of the five validated lncRNAs in the microarray and RT- qPCR data; the bars represent standard errors. ** statistically significant at: *p* < 0.01 (two-tailed *t*-test); *** statistically significant at: *p* < 0.001 (two-tailed *t*-test).

**Figure 5:**
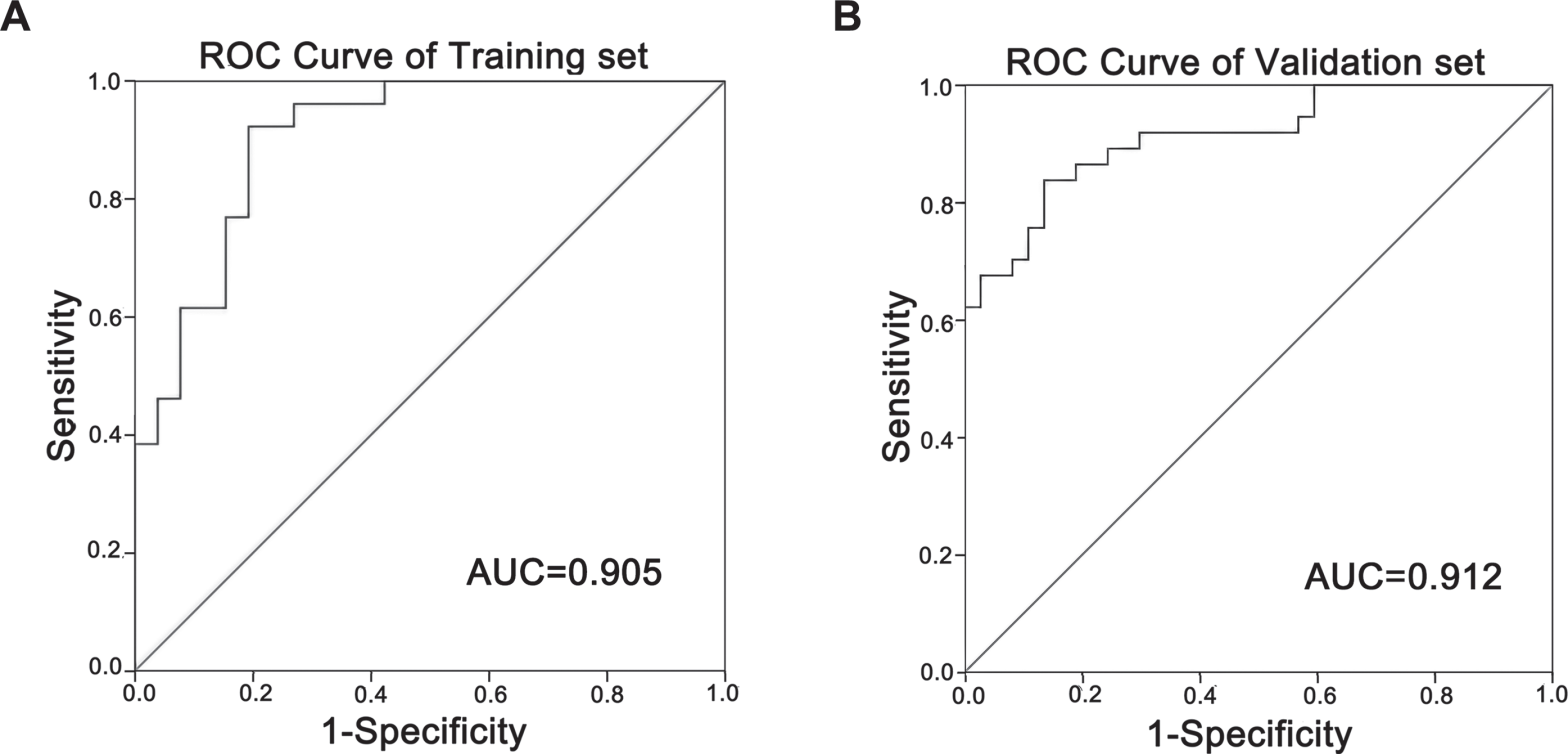
ROC curve analysis ROC curves of the panel in training set (**A**) and validation set (**B**) for the discrimination of LSCCs from NT samples. AUC: area under the curve.

In order to evaluate the diagnostic potential of lncRNAs in early stage LSCC, we adopt an established method to select candidate biomarkers from 2,668 DE-lncRNAs [16]. 16 upregulated lncRNAs and 10 downregulated lncRNAs were selected (Supplementary Table 3). Quantitative polymerase chain reaction (RT-qPCR) was used to further validate the expression levels of the 26 candidate lncRNAs in a training set of 24 pairs of early stage LSCC and NT samples (Table 1). Among these 26 candidates, five were validated which had significantly different expressions in the early stage LSCC samples (Table 2, Figure 4A–4E), while the other 21 candidates had no significant differences (Supplementary Table 4). These five lncRNAs showed the same trends in regulatory direction as the microarray data (Figure 4F), which supports a strong consistency between the qPCR and the microarray data. Then, ROC curve analysis was used to evaluate the distinguish capability of these five lncRNAs (Table 2). Combination of these five lncRNAs produced a higher AUC value (0.905, Figure 5A) with corresponding increases in sensitivity (92%) and specificity (83%).

**Table 1:** Clinical pathologic characteristics of the training set of 24 LSCC patients and the independent validation cohort of 39 LSCC patients

	Training set (24 pairs)	Validation set (39 pairs)
**Age**		
Mean ± SD	59.8 ± 5.6	61.5 ± 6.9
**Gender**		
Female	2	2
Male	22	37
**TNM stage**		
I	11	13
II	13	10
III	0	16
**Tumor grade (differentiation)**		
Well	5	4
Moderate	17	26
Poor	2	9

**Table 2:** Expression levels of the five lncRNAs and their AUC values in training set and validation set

Group	LncRNA	Mean (SD)in NT	Mean (SD)in LSCC	*p*-Value	AUC (SE)	Sensitivity(%)	Specificity(%)
**Training Set**:	ENST00000453324	1.19 (1.63)	3.74 (3.94)	0.001481	0.76 (0.07)	69	84
	NR_028500	0.98 (1.00)	2.36 (2.17)	0.001293	0.70 (0.07)	73	65
	UC011CLY.2	3.37 (3.36)	9.08 (5.85)	0.000013	0.77 (0.06)	84	66
	NR_046326	2.16 (2.44)	6.91 (5.40)	0.000040	0.79 (0.06)	84	74
	ENST00000441841	4.78 (5.20)	13.48 (11.01)	0.000288	0.76 (0.06)	73	73
**Validation Set**:	ENST00000453324	2.02 (2.18)	5.42 (4.49)	0.000010	0.77 (0.05)	64	80
	NR_028500	2.32 (2.66)	6.41 (6.16)	0.000191	0.73 (0.06)	75	68
	UC011CLY.2	3.94 (4.02)	12.64 (10.02)	0.000003	0.80 (0.05)	73	80
	NR_046326	4.12 (4.53)	10.65 (8.36)	0.000026	0.77 (0.05)	79	71
	ENST00000441841	8.10 (10.80)	20.10 (16.92)	0.000006	0.80 (0.07)	86	70

*Abbreviations: AUC: area under the receiver operating characteristic curve; SD: standard deviation; SE: standard error. Both the p value and AUC were obtained using the β-actin normalized values*.

We then used another independent set of 39 pairs of LSCC and NT samples (Table 1) to validate these five lncRNAs. Results yielded a similar result as training set (Table 2). Combination of five lncRNAs showed an AUC value of 0.912 (Figure 5B) with 85% sensitivity and 87% specificity. We also examined the expression levels of these five lncRNAs in advanced stage LSCC samples, the results showed that the differences were significant between NT and advanced stage LSCC samples, but were not significant between early and advanced stage LSCC samples (Supplementary Tables 5 and 6).

## DISCUSSION

People diagnosed with clinical stage I and II LSCC have a 60% to 40% 5-year rate of survival, however those with clinical stage III or IV disease have a less than 5% 5-year rate of survival [21]. Thus, early diagnosis may help to improve the 5-year rate of survival. A thorough understanding of the molecular aberrations of LSCC would assist in the biomarkers identification and early detection. Using a high-throughput lncRNA array, we identified thousands of DE-lncRNAs and DE-mRNAs in three pairs of early stage LSCC and corresponding adjacent NT samples. The potential functions of DE-lncRNAs were then discussed through function analysis and co-expression analysis, which were mainly based on the involvement of lncRNAs’ functions with their associated protein-coding genes [18, 22–25]. Results indicated that part of these DE-lncRNAs might be involved in some cancer-related pathway, but further studies were still needed to validate these speculations.

Increasing evidences have suggested that cancer-associated lncRNAs have the potential as diagnostic and prognostic biomarkers [26–28]. We then evaluated the diagnostic ability of lncRNAs in early stage LSCC. Using a training and validation set in a separate independent cohort, a panel of five lncRNAs was identified and optimized. Combination of these five lncRNAs shows a considerable power in distinguishing early stage LSCC samples from NT samples. The functions of these five lncRNAs remain unknown, however their associated coding genes may be involved in some cancer-related processes. For example, intron sense overlapping lncRNA ENST00000453324, also named as LINC000969, is located on the sense strand of chromosome 3 and is transcribed from the intron area of gene mucin 20 (MUC20). Overexpression of MUC20 can enhance EGF-induced phosphorylation of EGFR and STAT3 [29]. However, we didn't observe the aberrant expression of MUC20 in our microarray data, indicating the function of LINC000969 may not related to gene MUC20. NR_028500 is located on the sense strand of chromosome 11 and is a transcript variant of gene lactate dehydrogenase A (LDHA). Interestingly, NR_028500 associated gene LDHA is also found overexpressed in LSCC tissues (fold change > 2, *P* < 0.001). Protein encoded by this gene catalyzes the conversion of L-lactate and NAD to pyruvate and NADH in the final step of anaerobic glycolysis. Importantly, LDHA is upregulated in tumors and promotes cellular proliferation and tumorigenesis [30–32]. The co-expression of NR_028500 and LDHA implies that NR_028500 may be involved in the anaerobic glycolysis of tumor cells. ENST00000441841 and NR_046326 are two enhancer-like lncRNAs, which may increase transcription of their nearby coding genes [33]. NR_046326, also named as PCAT6 (prostate cancer associated transcript 6), is located on the sense strand of chromosome 1, and is first reported highly expressed in prostate cancer [34]. Study revealed that PCAT6 might enhance the expression of its neighbor gene KLHL12 and activate the Wnt–ß catenin pathway indirectly [33]. Recent studies identified that PCAT6 was significantly upregulated in lung adenocarcinoma tissues [35], and was positively associated with metastasis and cellular proliferation in lung cancer [36]. In this work, overexpression of PCAT6 was also identified in early stage LSCC tissues, implying that PCAT6 was closely related to the tumorigenesis in LSCC. LncRNA ENST00000441841 is located on the sense strand of chromosome X. Gene PLAC1 (placenta-specific protein 1 precursor) is on the upstream of it and defined as a neighbor coding gene (distance < 300 kb). PLAC1 is expressed exclusively in trophoblast cells in the mammalian placenta, and is involved in trophoblast invasion and migration [37]. Recent studies have detected high PLAC1 expression levels in a numbers of human solid tumors, including NSCLCs [38]. In our study, we also found the significant overexpression of PLAC1 (fold change > 90, *P* < 0.001) in LSCC tissues. These findings above suggest that ENST00000441841 may be involved in the activation of onco-placental gene PLAC1. Uc011cly.2 is transcribed from the sense strand of chromosome 5 and is overlapping with the transcript of gene SLC9A3 (solute carrier family 9 member a3). As a nature antisense lncRNA [25, 39], uc011cly.2 may be involved in the regulation of target gene SLC9A3 and its product. In summary, the associated genes mentioned above may offer clues for the function study of these five lncRNA biomarkers, however, further studies are still needed to illuminate their roles in the cancer processes. Collectively, our study is the first study that evaluate the potential of lncRNAs as biomarkers in early stage LSCC, and also provide important resource for further biological researches.

## MATERIALS AND METHODS

### Study samples

Paired LSCC and adjacent NT samples were collected from 63 patients (Table 1) who underwent primary surgical resection between August 2014 and September 2015 at Shanghai Zhongshan Hospital (Shanghai, China). The early stage LSCC in our study is defined as LSCC before stage III. No tumor metastasis was observed at the time of resection. All patients gave informed consent prior to sample collection. The pathological stage, grade, and nodal status was evaluated by an experienced pathologist and her colleagues. The patient's stage was determined according to the 7th edition of the TNM Classification of Malignant Tumors [40]. This study was approved by the Institutional Review Board of Shanghai Zhongshan Hospital.

### RNA extraction

Paired LSCC and adjacent NT samples from each subject were snap-frozen in liquid nitrogen immediately after resection and stored at –80°C until later use. Total RNA was isolated from the frozen tissues sample using All repo DNA/RNA Mini Kit (QIAGEN, Valencia, CA, USA) according to the manufacturer's instructions. Total RNA from each sample was quantified using a NanoDrop ND-1000 (Nano-Drop Technologies, Wilmington, DE, USA), and RNA integrity was assessed using standard denatured agarose gel electrophoresis.

### LncRNA and mRNA microarray expression profiling

Three pairs of early stage LSCC samples from 63 total were selected for lncRNA microarray analysis using an Arraystar Human lncRNA Microarray V3.0 (Arraystar, Rockville, MD, USA). The GEO accession number is GSE88862. Sample preparation and microarray hybridization was performed according to the manufacturer's standard protocols with minor modifications (Supplementary Method). After the hybridization and array image acquisition, quantile normalization and subsequent data processing were performed using the GeneSpring GX v11.5.1 software package (Agilent Technologies, Santa Clara, CA, USA). The *p*-values were adjusted through Benjamini-Hochberg method. The threshold used to determine up- or down-regulated lncRNAs and mRNAs was a fold-change > 2.0 and FDR < 0.05. All microarray work was performed by Kangchen Bio-Tec (Shanghai, P.R. China).

### Co-expression and functional group analysis

Co-expression analysis was performed by associating the expression profiles of DE-lncRNAs with DE-mRNAs [41]. This association was based on the information of the lncRNAs’ associated coding regions, which was supplied by microarray analysis. GO analysis was derived from Gene Ontology (http://www.geneontology.org). We used GO analysis to associate differentially expressed mRNA with GO functional categories. We also performed a pathway analysis for the differentially expressed mRNA based on the latest KEGG (Kyoto Encyclopedia of Genes and Genomes) database. The *p-value* (Hypergeometric-*P value*) denoted the significance of the pathway correlated to the conditions. Recommend *p-value* cut-off (0.05) was used.

### Selecting candidate biomarkers

LncRNA was chosen for further selection if its raw expression intensity was between 100 and 20,000 in all three pairs of LSCC samples. The average raw expression intensity across all three tumor samples (T ~) and nontumorous samples (N ~) was then calculated. The difference between T ~ and N ~ was denoted as A=|T ~-N ~|. N_max_ and N_min_ represents the maximum and minimum raw intensity in NT samples respectively; T_max_ and T_min_ represents the maximum and the minimum raw intensity in LSCC samples respectively. A given lncRNA was selected as a candidate biomarker if either N_max_ − N_min_ or T_max_ − T_min_ was smaller than A/10.

### Quantitative PCR

Complementary DNA (cDNA) was reverse transcribed and synthesized using the SuperScript III First Strand Synthesis System (Invitrogen, Carlsbad, CA, USA) according to the manufacturer's instructions. We added approximately 1 μg of total RNA to each reaction. The RT-qPCR was performed on a Light Cycler 480 (Roche Diagnostics, Penzberg, Germany) using LightCycler 480 SYBRGreen I Master (Roche Diagnostics, Mannheim, Germany). The thermocycling protocol included the following steps: denaturation step of 10 min at 95°C; 40 cycles of 95°C for 15 s, and 60°C for 1 min. after the amplification, a melting curve analysis was performed at 95°C for 5 s and 65°C for 1 min to monitor primer dimers or nonspecific product formation. Each sample was measured in triplicate and a negative control was included with every RT-qPCR assay. β-actin was used as the reference gene instead of GAPDH since a recent study reported that the latter had differential expression between LSCC and NT samples [42]. Relative expression levels were quantified based on the crossing point values and normalized to the reference gene. The gene expression levels were calculated by the 2^-ΔΔCp^ method. The primer sequences used in the RT-qPCR are listed in Supplementary Table 7.

### Statistical analysis

SPSS version 22.0 software (SPSS, Inc., Chicago, IL) was used for all statistical analyses. Student's *t-test* was used to analyze the lncRNA expression differences between samples of LSCC and adjacent NT samples. ROC curves were constructed to discriminate between the two different kinds of samples. The AUC value was used to assess for predictive power. The sensitivity and specificity were calculated according to the standard formulas.

## SUPPLEMENTARY MATERIALS TABLES


