# Supplementary material for: Identification of long noncoding RNAs for the detection of early stage lung squamous cell carcinoma by microarray analysis
Source: Oncotarget. 2017 Jan 5;8(8):13329–37. doi: 10.18632/oncotarget.14522 (PMC5355100; doi:10.18632/oncotarget.14522)
Supplement: Supplementary file 1 [file oncotarget-08-13329-s001.pdf]

Identification of long noncoding RNAs for the detection of early stage lung squamous cell carcinoma by microarray analysis

Supplementary Materials

Procedure of sample preparation and microarray hybridization

Briefly, mRNA was purified from total RNA after removal of rRNA using mRNA-ONLYTM Eukaryotic mRNA Isolation Kit (Epicentre, Madison, WI, USA). Then, each sample was amplified and transcribed into fluorescent cRNA using a random priming method along the entire length of the transcripts without 3′ bias. The labeled cRNA was then purified using an RNeasy Mini Kit (QIAGEN, Valencia, CA, USA) and hybridized onto the Human LncRNA Array v3.0 (8 × 60 K, Arraystar). After washing the slides, the arrays were scanned by an Agilent Scanner G2505C (Agilent Technologies, Santa Clara, CA, USA). The acquired array images were analyzed using Agilent Feature Extraction software (version 11.0.1.1).

Supplementary Table 1: Top 15 enriched GO terms in the co-expressed DE-mRNAs groups

category	down-regulated	up-regulated
**CC**	extracellular space	nucleoplasm
**CC**	extracellular exosome	nuclear chromosome
**CC**	clathrin-coated endocytic vesicle	nucleosome
**CC**	lamellar body	nucleus
**CC**	proteinaceous extracellular matrix	nuclear chromosome, telomeric region 9
**MF**	IgG binding	protein heterodimerization activity
**MF**	growth factor binding	DNA binding
**MF**	glutathione transferase activity	DNA binding, bending
**MF**	serine-type endopeptidase inhibitor activity	cofactor binding
**MF**	peptide hormone binding	RNA polymerase II transcription factor activity, sequence-specific DNA binding
**BP**	regulation of complement activation	cartilage development
**BP**	regulation of bone resorption	telomere organization
**BP**	positive regulation of epithelial cell migration	nucleosome assembly
**BP**	respiratory gaseous exchange	dTTP biosynthetic process
**BP**	positive regulation of ERK1 and ERK2 cascade	response to cadmium ion

Supplementary Table 2: Top 8 enriched pathways in the co-expressed DE-mRNAs groups

Up-regulated	Down-regulated
Cell cycle	Phagosome
Alcoholism	Drug metabolism - cytochrome P450
Systemic lupus erythematosus	Complement and coagulation cascades
Transcriptional misregulation in cancer	Pertussis
Viral carcinogenesis	Staphylococcus aureus infection
Choline metabolism in cancer	Leishmaniasis
Glutathione metabolism	Metabolism of xenobiotics by cytochrome P450
Small cell lung cancer	Chemical carcinogenesis

Supplementary Table 3: 26 selected lncRNAs as candidate biomarkers

Seqname	Gene Symbol	Regulation	*P*-Value	FDR	Fold Change
**ENST00000579581**	RP11-697E2.7	UP	6.207E-08	0.000014103	**15.0330204**
**TCONS_00029159**	XLOC_014103	UP	8.3329E-07	0.000070817	**15.5742163**
**ENST00000453324**	SDHAP2	UP	2.41806E-06	0.00012018	**3.1505568**
**ENST00000505930**	RP11-542G1.1	UP	4.05634E-06	0.000158687	**5.3993704**
**ENST00000393515**	ANKRD36BP2	UP	7.35201E-06	0.000217686	**5.7717817**
**UC021VCR.1**	BC011243	UP	1.37871E-05	0.000323142	**3.5134994**
**NR_028500**	LDHA	UP	2.0971E-05	0.000420132	**2.2094189**
**ENST00000444125**	RP11-65J3.1	UP	2.36616E-05	0.000445108	**3.2391902**
**ENST00000512916**	HOXC-AS5	UP	2.43353E-05	0.000451429	**3.56718**
**ENST00000567753**	RP11-524C21.2	UP	2.61408E-05	0.000466387	**6.2628484**
**UC003TTF.3**	BC053669	UP	3.39083E-05	0.000530469	**3.9300781**
**UC011CLY.2**	BC013821	UP	6.61701E-05	0.00077626	**2.1427106**
**ENST00000423466**	RP11-114M1.1	UP	9.70798E-05	0.000982277	**4.1091657**
**ENST00000448923**	RP11-96L14.7	UP	0.000317504	0.002129383	**2.1227999**
**NR_046326**	KDM5B-AS1	UP	0.000353536	0.002296335	**2.2237249**
**ENST00000441841**	RP3-473B4.3	UP	0.000960839	0.00471858	**3.0955763**
**ENST00000534789**	NAPSB	DOWN	6.39371E-06	0.000201077	**3.7887922**
**NR_037595**	HHIP-AS1	DOWN	7.15453E-06	0.000214232	**4.6442937**
**ENST00000420160**	U82670.4	DOWN	8.57717E-06	0.000240528	**8.0246782**
**NR_024065**	LINC00312	DOWN	2.21674E-05	0.000433451	**6.0600542**
**ENST00000445003**	RP11-290F20.3	DOWN	2.54095E-05	0.000460723	**7.100801**
**ENST00000568248**	AC083843.1	DOWN	3.82868E-05	0.000566817	**3.468433**
**ENST00000557532**	DIO3OS	DOWN	6.4091E-05	0.000759877	**2.4353256**
**ENST00000507983**	APOC1P1	DOWN	7.22899E-05	0.00081789	**5.1270217**
**ENST00000556266**	DIO3OS	DOWN	9.80552E-05	0.000989411	**3.1239347**
**ENST00000440498**	AGAP1-IT1	DOWN	0.000151153	0.00129845	**2.5613727**

Supplementary Table 4: q-PCR results of 21 lncRNA candidates whose *p*-values are > 0.05

LncRNA	Mean(SD) in NT	Mean(SD) in LSCC	*P*-Value
**ENST00000579581**	1.559012 (2.327780)	1.609670 (1.869540)	0.909214
**TCONS_00029159**	0.608578 (0.859318)	0.698612 (0.790943)	0.667664
**ENST00000505930**	2.962579 (3.943876)	4.785218 (4.671481)	0.069069
**ENST00000393515**	38.815625 (58.396375)	80.868110 (105.207333)	0.087900
**UC021VCR.1**	0.865324 (1.478653)	0.881623 (0.785582)	0.944659
**ENST00000444125**	1.734500 (1.834496)	3.906844 (5.907817)	0.111038
**ENST00000512916**	4.019584 (2.826905)	3.656126 (2.819002)	0.240873
**ENST00000567753**	3.407716 (4.237897)	2.268392 (2.216529)	0.245398
**UC003TTF.3**	1.044625 (1.176549)	0.672548 (0.655613)	0.175170
**ENST00000423466**	4.626184 (6.182668)	5.307664 (7.017111)	0.410595
**ENST00000448923**	59.022878 (120.387290)	71.603390 (224.073974)	0.663378
**ENST00000534789**	0.946333 (1.191640)	0.984365 (1.010623)	0.902521
**NR_037595**	1.786038 (3.018431)	4.757554 (17.991131)	0.388400
**ENST00000420160**	1.326469 (1.310435)	1.780472(1.340934)	0.277118
**NR_024065**	2.976263 (3.839094)	5.819243 (15.696177)	0.338054
**ENST00000445003**	1.189236 (1.183239)	1.163785 (1.486483)	0.936000
**ENST00000568248**	1.242926 (1.652666)	1.207177 (1.715762)	0.929575
**ENST00000557532**	2.941192 (4.106672)	2.742018 (4.410143)	0.790469
**ENST00000507983**	5.193950 (8.461362)	3.450538 (4.315370)	0.296442
**ENST00000556266**	1.537714 (3.269819)	1.229034 (1.650816)	0.541172
**ENST00000440498**	0.354999 (0.250134)	0.364788 (0.286465)	0.836739

Supplementary Table 5: Expression levels of the 5 lncRNAs between early stage samples and advanced stage samples

LncRNA	Mean(SD) in early stage	Mean(SD) in advanced stage	*p*-value
**ENST00000453324**	2.970(2.239)	2.090(2.264)	0.252
**NR_028500**	3.726(3.452)	2.151(1.974)	0.081
**UC011CLY.2**	6.442(5.135)	5.711(4.998)	0.672
**NR_046326**	6.004(4.537)	3.802(3.185)	0.089
**ENST00000441841**	10.698(8.926)	9.431(8.000)	0.664

Supplementary Table 6: Expression levels of the 5 lncRNAs between NT samples and advanced stage LSCC samples

LncRNA	Mean(SD) in NT	Mean(SD) in advanced stage LSCC	*p*-value
**ENST00000453324**	0.698262 (0.863966)	2.090018 (2.264368)	0.035716
**NR_028500**	0.591525 (0.476840)	2.150529 (1.974601)	0.006987
**UC011CLY.2**	2.214971 (2.745987)	5.711486 (4.998583)	0.019408
**NR_046326**	1.854868 (1.882079)	3.802167 (3.185107)	0.041495
**ENST00000441841**	3.676148 (4.655389)	9.431781 (8.000265)	0.003569

Supplementary Table 7: primers sequences used for RT-qPCR

Seqname	Forward primer sequence	Reverse primer sequence
**ENST00000579581**	TCCTGACCCTGCTCCTACTC	GGAAGGAAACATCTCCACCAC
**TCONS_00029159**	ATGGGGAGGATGAGGTTTG	CCGTCTTGAGGCAGGTGTT
**ENST00000453324**	GTGTGTGTGAGTATGTGACGGAG	CACACGCCGACTTCAGGTT
**ENST00000505930**	GCAGAACAGCAAAGATGGC	TTGACAGACACCTCACGCA
**ENST00000393515**	TTTGTAGCCGACGATTGTG	CGGATGCTTTCCTCCTTGT
**uc021vcr.1**	CACCCAGATAGCCTCACAA	TAAGGCTTTCAGGGTAGGC
**NR_028500**	GCCATCAGTATCTTAATGAAGACT	AACCGCTTCCAATAACACG
**ENST00000444125**	AATGGAGTCGCTCTGGTTC	TGTTGCTACTGTGGTTGGG
**ENST00000512916**	CTCGCTCAGCCAGACCTACT	CAACCGTCAAACACCACCTTC
**ENST00000567753**	ATTGATGGAGGGACCGTGG	CTGTGTGGGGTTCAGGGCT
**uc003ttf.3**	TGAGACGCCTCTGTTTACCTT	GGGCTTTTTCCTTCATTACG
**uc011cly.2**	GCCTGCTCTTTTGCTTCTAA	CCTCACGTCTGTCTTCGTG
**ENST00000423466**	AGAATGTGTGAAAGCAGCAAC	GCGAATGTAAGCAAAGAACC
**ENST00000448923**	TGCTATGCCGTCTGGAAGA	GATTGATGGATGGGTGCTC
**NR_046326**	CTTTCCCTCGTCCTCTGCG	GCAAGCGTTTGTGGGTTTCA
**ENST00000441841**	GCCTTCCATCTGACACTCCT	TTCTGTTCCCCCTTCTTACC
**ENST00000534789**	CCCTCTTCGTCAAGTCCAC	GAGGAGCCAGTGTCAAAGG
**NR_037595**	GAGTGGTGAAAAGAAGAAGGG	GGTGGTGTGAGCAGAAGATGT
**ENST00000420160**	CATTCCCCGACACACCCAG	TCTGTCCGTGGTTCCTGCC
**NR_024065**	TAAGGAGACAAGCGAACCAAG	AAGCAGTCCAGAGGTTAGTTCAC
**ENST00000445003**	TGGAGCAGAGCCAGTGGAA	CAGTGGAGGCAGAAGGAAGG
**ENST00000568248**	CTGTCGGATCAGTGGCAGC	CACCTCAACTCATCGGGCA
**ENST00000557532**	AGGGCTGTCATCTGTTCTGG	CCGAGGTGTGGAGTGTATTCT
**ENST00000507983**	AAGGATTCAGGTTGGTGCC	CACTCTGTGTGATGCGGTTG
**ENST00000556266**	TAGGGCTGTCATCTGTTCTGG	CGAGGTGTGGAGTGTATTCTGT
**ENST00000440498**	CCGCTTGCTGAGTCTTTCT	GAGGTCCTAAGTCAGGGTCG
**β-ACTIN**	CCTGTACGCCAACACAGTGC	ATACTCCTGCTTGCTGATCC
